# Biokinetic modelling of the exhalation of ^219^Rn gas and its airborne progeny from patients undergoing treatment with ^223^Ra-dichloride and effective dose estimation for caregivers

**DOI:** 10.1007/s00411-025-01193-5

**Published:** 2026-05-18

**Authors:** Lena Katzdobler, Astrid Delker, Augusto Giussani, Kerstin Hürkamp, Christina Kuttler, Oliver Meisenberg, Sibylle Ziegler, Wei Bo Li

**Affiliations:** 1 Department of Medical and Occupational Radiation Protection, Federal Office for Radiation Protection, 85764 Oberschleißheim, Germany; 2 Department of Mathematics, Technical University of Munich, 85748 Garching, Germany; 3 Department of Nuclear Medicine, LMU University Hospital, LMU, 81377 Munich, Germany; 4 Department of Environmental Radioactivity, Federal Office for Radiation Protection, 85764 Oberschleißheim, Germany; 5 Department of Nuclear Medicine, Hannover Medical School, 30625 Hannover, Germany

**Keywords:** ^219^Rn, ^223^RaCl_2_, Radionuclide therapy, mCRPC, Alpha-particle emitters, Exhalation, Biokinetic modelling, Effective dose

## Abstract

The radiopharmaceutical ^223^Ra-dichloride (Xofigo^®^) has been approved for palliative treatment of metastatic castration-resistant prostate cancer. During the treatment and caregiving, patients who received ^223^Ra-dichloride can exhale the progeny ^219^Rn, a radioactive gas. This causes a potential risk for medical staff and caregivers who might inhale the ^219^Rn gas and its airborne decay products. As an addition to the strategies of developing devices for measuring ^219^Rn gas and its daughter nuclides in treatment rooms, this work tried to estimate the amount of exhaled ^219^Rn and of its airborne progeny using biokinetic modelling. The biokinetic models and parameters for ^223^Ra and its progeny ^219^Rn, as published by the International Commission of Radiological Protection, were adopted for this analysis. Without consideration of room ventilation, the model predicted that the amount of ^219^Rn gas in the room air can reach a maximum activity of 5.8∙10^− 2^ Bq at approximately 1.5 min after infusion of one Bq of ^223^RaCl_2_. The summed activities of ^219^Rn and its airborne progeny do not exceed 1.2∙10^− 1^ Bq per infused Bq of ^223^RaCl_2_. In the breathing air of the patients, the activities at 1 and 5 min after the infusion were estimated to be approximately 2∙10^− 2^ Bq and 1.8∙10^− 2^ Bq per Bq of ^223^RaCl_2_, respectively. The values at 1 min are comparable to the measured results reported in the literature. The effective dose from inhalation of ^219^Rn gas and its airborne progeny due to a treatment of 4015 kBq of ^223^RaCl_2_, was estimated to be 232.6 nSv for medical staff and 7.5 µSv for caregivers.

**Supplementary Information:**

The online version contains supplementary material available at 10.1007/s00411-025-01193-5.

## Introduction

 Prostate cancer is the most common kind of cancer in men in Europe, with 10–20% of patients developing a castration resistant form (Bray et al. 2018). After the clinical trials, Radium-223 dichloride (^223^RaCl_2_), marketed under the brand name Xofigo^®^, has been approved in recent years for the palliative treatment of metastatic castration-resistant prostate cancer (Parker et al. 2013; EMA 2018). Radium-223 is a bone-seeking calcium mimetic, which allows it to target areas with increased bone turnover, especially regions carrying bone metastases (Parker et al. 2013). As an alpha-emitter, it releases its energy in very small volumes, with a range of less than 100 μm (Parker et al. 2013). This achieves a tightly localized cytotoxic effect on the tumour cells and consequently minimizes the extent of damage to the surrounding healthy tissue (Suominen et al. 2017; Nilsson 2015). Administered via an injection or an infusion pump, ^223^RaCl_2_ enters the bloodstream of the patient, distributes throughout the body and, from there, is either excreted or decays directly to the radioactive ^219^Rn gas.

**Fig. 1:**
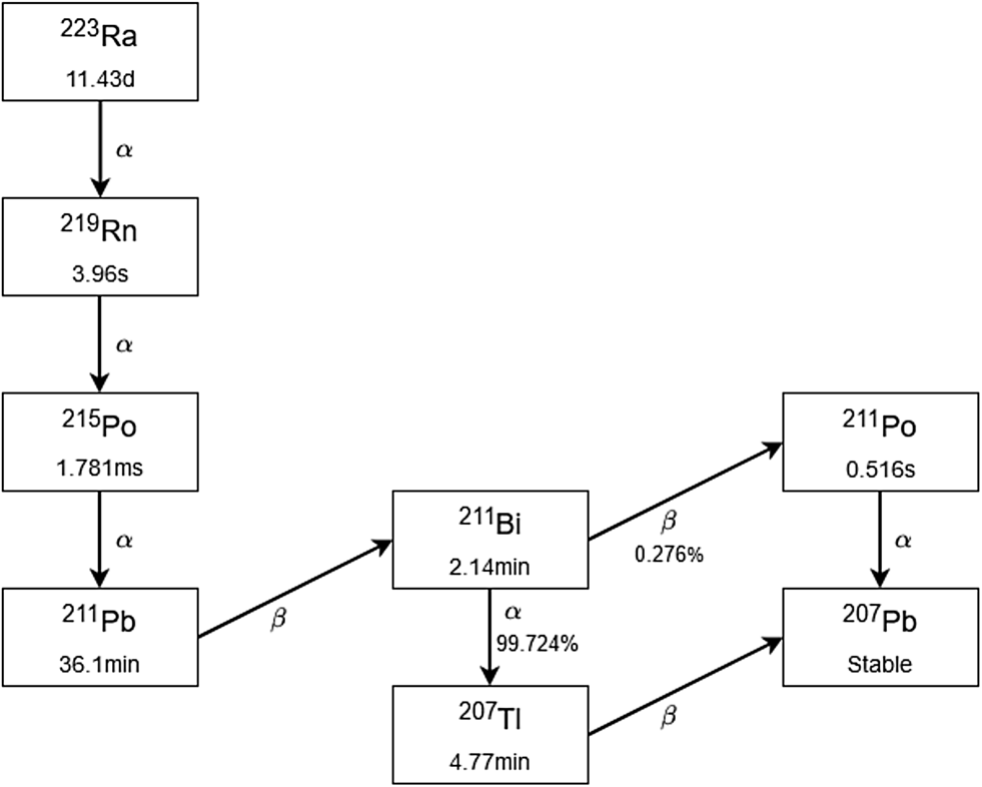
Decay chain of ^223^Ra

In addition to assessing the absorbed dose to patients receiving ^223^RaCl_2_ (Höllriegl et al. 2021), radiation protection of medical staff and caregivers during the treatment and healthcare is a standard procedure in nuclear medicine departments (Hosono et al. 2019). As a noble gas, radon is highly volatile and can escape the patient’s body quite rapidly. According to ICRP, much of the gas in the blood is cleared by the lungs in a single pass, causing the patient to exhale ^219^Rn gas (ICRP 2017). Radon-219 and its radioactive decay products (^215^Po, ^211^Pb, ^211^Bi, ^207^Tl, ^211^Po, see Fig. 1) pose a potential risk of radiation exposure to clinical staff and caregivers present in the patient’s room.

Yamamoto et al. (2018) compared the concentration of ^222^Rn and daughter radionuclides of ^219^Rn and ^222^Rn, in presence and absence of patients during ^223^Ra radionuclide therapy. They demonstrated for the first time that the concentration of radon progeny in the room air increased up to 4 to 5 times when the patient was in the room. This was done by measuring the energy peak (6.6 MeV) of the progeny ^211^Bi shown in the energy spectra of the alpha-particles. Nagata et al. (2020) predicted that the dispersal rate of ^219^Rn from an aqueous ^223^Ra solution containing sodium chloride/citrate, whose composition is chemically identical to Xofigo^®^, would be below 1.6∙10^− 3^ h^− 1^. The release of ^219^Rn gas from the syringe into the treatment room air is considered to be negligible. Nevertheless, a mask should be worn during the syringe preparation and administration (Deshayes et al. 2017; Bolin and Groves 2025).

The therapy with Xofigo^®^ is ambulatory and therefore the patient is free to leave the hospital after the treatment (Dauer et al. 2014; Higashi 2018; Parker et al. 2018). Wanke et al. (2021) measured the external exposure of relatives and caregivers from the patients 10–20 min post injection (p.i.) to be less than 0.080 µSv h^− 1^ in median at 1 m distance. Through saliva samples collected 20–30 min and 3–4 h p.i., they found a median of 27 Bq g^− 1^ and 14.7 Bq g^− 1^ of ^223^Ra, respectively. Additionally, sweat samples taken within the first 24 h p.i. yielded a median value of 0.121 Bq cm^− 2^. The contamination levels in restrooms and kitchens were low, with median values ranging from 2.6∙10^− 5^ to 2.15∙10^− 4^ Bq cm^− 2^.

Overall, the literature suggests that a significant portion of the elevated activity concentration levels in the room air during ^223^RaCl_2_ therapy results from exhalation of ^219^Rn gas by patients. Since ^219^Rn gas has a very short half-life of only 3.96 s, it is extremely difficult to directly measure its exhaled amount present in the room air. Wanke et al. (2019) attempted to measure ^219^Rn gas with a commercial radon monitor, the Alphaguard (Saphymo, formerly Genitron, Frankfurt, Germany), and concluded that this instrument cannot detect ^219^Rn gas at concentrations below 600 kBq m^− 3^. In one of their latest studies, Wanke et al. (2025) aquired an expected value for the activity concentration at 20–30 min p.i. of 6 kBq L^− 1^ (6 MBq m^− 3^) using the Alphaguard. At 3–4 h p.i., they measured a mean activity concentration of 4.4 kBq L^− 1^ (4.4 MBq m^− 3^). An alternative method of measuring ^219^Rn gas is to measure one of its progeny with a comparatively long half-life. Ooe et al. (2019) collected breath samples from patients undergoing treatment with Xofigo^®^ and measured the activity of the progeny ^211^Bi using gamma spectrometry with an HPGe detector and thereafter retrospectively estimated the ^219^Rn activity. These measurements showed considerable inter-patient variation, with ^219^Rn gas activities at 1 min between 3.7∙10^− 3^ and 2.4∙10^− 2^ Bq per infused Bq of ^223^RaCl_2_.

Lassmann and Nosske (2013) used the radium model from Publication 67 of the International Commission of Radiological Protection (ICRP) (ICRP 1993) to calculate organ absorbed doses during ^223^Ra-chloride treatment. Recently, ICRP Publication 137 (ICRP 2017) provided a detailed description of the updated models for the systemic biokinetics of radium absorbed into blood after inhalation and ingestion, as well as its progeny produced within the body. Höllriegl et al. (2021) applied this model for pharmacokinetic modelling and dosimetry for ^223^RaCl_2_.

The goal of this study was to predict the activity in the breath of patients undergoing Xofigo^®^ treatment and in the room air of medical treatment and patient rooms. The results were used for the estimation of the radiation exposure of medical staff and caregivers by calculating the corresponding effective doses. To achieve this, biokinetic models in the form of compartmental structures were constructed and evaluated. In a similar way to the modelling work of Lassmann and Nosske (2013) and Höllriegl et al. (2021), this study adopted the ICRP systemic biokinetic population model of radium and its progeny ^219^Rn for occupationally exposed persons (ICRP 2017) to model the exhaled ^219^Rn gas and its airborne progeny from patients receiving ^223^RaCl_2_ administration.

In order to critically evaluate the radium model of ICRP Publication 137 (ICRP 2017) as a choice for the modelling of ^223^RaCl_2_, in the Annex, the predicted activities in organs after administration of ^223^Ra were compared to the description of the pharmacokinetic properties in the professional information of Xofigo^®^ (Bayer 2013) and to the results of two phase-I-studies (Yoshida et al. 2016; Carrasquillo et al. 2013).

## Materials and methods

In this section, the modelling according to the ICRP (ICRP 1995, 2006, 2017) and the dosimetry are described. Throughout the rest of this work, the term post infusion (p.i.) refers to “after the beginning of the infusion”.

### Systemic model of ^223^Ra

**Fig. 2:**
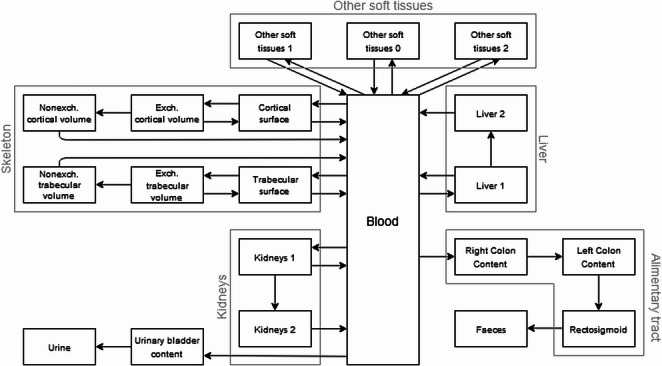
Biokinetic model of ^223^Ra according to the ICRP (ICRP 1995, 2006, 2017)

 The structure of the biokinetic model for radium is displayed in Fig. 2 and the corresponding transfer coefficients are listed in Table S1 in the supplementary material. The human alimentary tract model (HATM) and its transfer coefficients are described in ICRP Publication 100 (ICRP 2006). Usually, a reabsorption from the HATM into blood is assumed to take place and as a simplification, this reabsorption is only considered from the small intestine contents (ICRP 2015). However, because the systemic radium model expects no transfer of the radionuclide into the small intestine, no reabsorption from the HATM into blood is accounted for. The elimination rate from the bladder is assumed to be 12 d^− 1^ (ICRP 1995).

### Treatment of ^219^Rn gas as a progeny of ^223^Ra

**Fig. 3:**
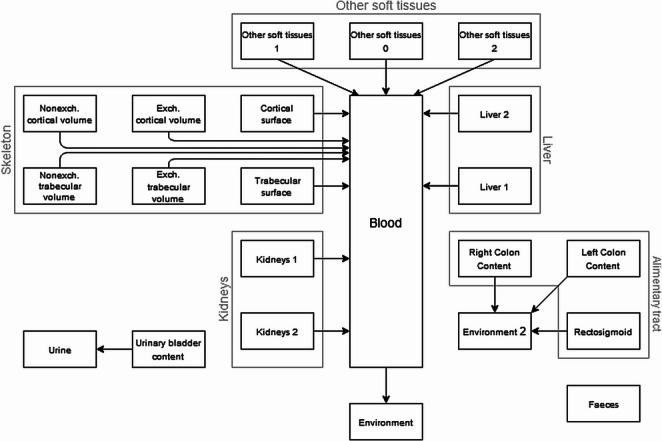
Modified compartmental model structure of ^219^Rn gas as a progeny of ^223^Ra (ICRP 1995, 2006, 2017)

 The compartmental model used by the ICRP to describe the systemic biokinetics of radon as a progeny of radium has the same structure as the radium model, but with different transfer pathways (ICRP 2017). Since radon is a chemically inert gas, no interaction between the compartments will take place, but the radionuclide will rather follow a strict excretion route. This means that the ^219^Rn gas in the systemic compartments will be absorbed into the blood stream and excreted via exhalation. Radon-219 in the alimentary tract is modelled to escape the body from the corresponding compartments to the environment, as recommended by the ICRP (ICRP 2015). In this work, the activity from the alimentary tract is transferred to the compartment “Environment 2”. Since there is no information available, ^219^Rn gas in the bladder is modelled to transfer to the compartment “Urine” with a reference excretion rate of 12 d^− 1^. There is no transfer assumed into the bladder and the HATM in the radon model, therefore the compartments for the bladder and the alimentary tract represent the direct decay products of ^223^Ra (according to Fig. 2). For the modelling of the exhalation, a simplification in the form of a direct removal from blood to the environment according to ICRP 137 (ICRP 2017) is applied, expecting no interaction of the gas with the respiratory tract. This is quite similar to the structure of the systemic biokinetic model of radon as a parent nuclide (ICRP 2017). The compartment “Environment” represents the activity of ^219^Rn gas in the room air exhaled by the patient. The structure of the ^219^Rn model as a progeny of ^223^Ra is shown in Fig. 3 with corresponding transfer coefficients in Table S2 in the supplementary material. The simulation itself only models the amount of exhaled ^219^Rn gas, but does not model how this amount in the room air is further influenced by additional factors such as room ventilation. These factors will be considered at a later stage during the estimation of the effective dose to caregivers and medical staff.

According to the systemic biokinetic models of the progeny of ^219^Rn described in ICRP 137 (ICRP 2017), the particles do not transfer from the blood to the respiratory tract. Therefore, they are not exhaled by the patient. This means, only the amount of ^219^Rn gas in the environment and its airborne progeny are of interest in this study and the biokinetic models of the progeny of ^219^Rn gas are not considered. Consequently, in the compartment model, only the decay of ^219^Rn is simulated in the body. For the ^219^Rn progeny, only the environmental compartments were explicitely modelled.

### Implementation in SAAM II

In order to evaluate the radioactivity in the room air, the ordinary differential equations (ODEs), that are defined by the compartment models described above, are solved numerically with the help of the commercial software SAAM II (Barrett et al. 1998; Perazzolo 2024). With this software, biokinetic models can be constructed either with the help of a compartmental module or a numerical module. In this study, the compartmental module was used. As exogenous input, a constant infusion over 1 min into the blood compartment was chosen, because this is the common duration of the infusion during the treatment with Xofigo^®^ according to the instructions in the professional information (Bayer 2013).

Within the biokinetic model and modelling of progeny in the environmental compartments, physical decay was always accounted for.

### Modelling the exhalation of ^219^Rn in the air

Ooe et al. (2019) collected breath samples after 1 min and 5 min p.i. of patients undergoing treatment with ^223^RaCl_2_. Based on measuring the photopeak of the daughter nuclide ^211^Bi, they calculated the activity of ^219^Rn exhaled by the patient at 1 min and at 5 min p.i. of 2.5 MBq, 3.2 MBq, 3.8 MBq, 3.9 MBq and 4.3 MBq of ^223^RaCl_2_. The simulated activity of ^219^Rn in the breath of the patient was compared to the results of Ooe et al. (2019) using the solution of the “Environment” variable of the ^219^Rn model. To define the exact time point for the comparison, a respiration frequency of 12 min^− 1^ was used in agreement with Ooe et al. (2019), leading to a duration of 5 s for one complete respiration event (ICRP 1994). Thus, for a realistic simulation of the measurements of Ooe et al. (2019), the time point of interest is 2.5 s after 1 min and after 5 min p.i., assuming that an exhalation takes up half the time of a full respiration.

### ^219^Rn activity escaped from the alimentary tract

The activity in the compartment “Environment 2” was evaluated for ^219^Rn gas and its airborne progeny to estimate the amount of activity leaving the patient’s body through the alimentary tract as recommended by ICRP 130 (ICRP 2015).

### Radiation exposure of medical staff and caregivers

#### Dosimetry of ^219^Rn gas

In addition to the measurements of exhaled ^219^Rn, Ooe et al. (2019) also estimated the radiation exposure to medical staff by calculating the effective dose due to inhalation of the exhaled ^219^Rn gas without consideration of its progeny. Based on the simulated amount of ^219^Rn gas in the room air in this study, the effective dose to caregivers was estimated as described below.

In the work of Ooe et al. (2019), the impact of the air changes on the ^219^Rn gas activity in the room air was represented by the division by the rate *N*_*ac*_ = 1 h^− 1^, assuming that the air is completely replaced every hour. In this work, a factor was chosen that considers complete mixing of the room air containing ^219^Rn with the supply air.

If the ^219^Rn gas concentration in the room is constant, the formula for the effective dose of ^219^Rn, *E*_*Rn*_, to a person through inhalation over a period of time *T* is

1 $$\:{E}_{{Rn}}=\frac{{\bar{A}}_{Rn}}{{{V}}_{{room}}}\cdot\:{{V}}_{{daily}}\cdot\:T\cdot\:{e}_{Rn}\cdot\:F,$$

where $\:{\bar{A}}_{Rn}$ is the exhaled ^219^Rn gas activity, *V*_*room*_ is the room volume, *V*_*daily*_ is the respiration rate, *e*_*Rn*_ is the effective dose coefficient of ^219^Rn and *F* is the exposure factor^1^ (Hosono et al. 2019).

In order to take a regular ventilation into account, a factor *n*_*Rn,ac*_ for the air change in the room needs to be added to Eq. (1), leading to

2 $$\:{E}_{Rn}=\frac{{\bar{A}}_{Rn}}{{{V}}_{{room}}}\cdot\:{n}_{Rn,ac}\cdot\:{{V}}_{{daily}}\cdot\:T\cdot\:{e}_{Rn}\cdot\:F.$$

Derivation of n_ac_:

The amount of ^219^Rn gas in the room air, *N*_*Rn,ac*_, under consideration of regular air changes in the room, changes according to the following equation (Meisenberg and Tschiersch 2010):

3 $$\:\frac{d{N}_{Rn,ac}}{dt}={\bar{A}}_{Rn}-{\lambda\!\!\:}_{Rn}\cdot\:{N}_{Rn,ac}-{N}_{ac}\cdot\:{N}_{Rn,ac},$$

where $\:{\lambda\:}\!\!_{Rn}$ is the decay constant of ^219^Rn and *N*_*ac*_ corresponds to the ventilation in the room. From the common formula $\:A\:=\:N\cdot\:\lambda\:$, Eq. (3) can be written as

4 $$\begin{aligned}\:{{\uplambda\:}}\!\!_{Rn}\cdot\:\frac{d{N}_{Rn,ac}}{dt}&={{\uplambda\:}}\!\!_{Rn}\cdot\:\left({\bar{A}}_{Rn}-{\lambda\:}\!\!_{Rn}\cdot\:{N}_{Rn,ac}-{N}_{ac}\cdot\:{N}_{Rn,ac}\right)\\ \Leftrightarrow\frac{d{A}_{Rn,ac}}{dt}&=\:{{\uplambda\:}}\!\!_{Rn}{\bar{A}}_{Rn}\:-{\lambda\:}\!\!_{Rn}\cdot\:{A}_{Rn,ac}-{N}_{ac}\cdot\:{A}_{Rn,ac}.\end{aligned}$$

In the assumption of a steady state, Eq. (4) yields

5 $$\begin{aligned}\:\:0&=\:{{\uplambda\:}}\!\!_{Rn}{\bar{A}}_{Rn}\:-{\lambda\:}\!\!_{Rn}\cdot\:{A}_{Rn,ac}-{N}_{ac}\cdot\:{A}_{Rn,ac} \\\:\Leftrightarrow{A}_{Rn,ac}&=\:{\bar{A}}_{Rn}\cdot\:\frac{{\lambda\:}\!\!_{Rn}}{{\lambda\:}\!\!_{Rn}+{N}_{ac}}.\end{aligned}$$

Therefore, the factor for the air changes is defined as

6 $$\:{{n}}_{{Rn,ac}}=\frac{{\lambda\!\!\:}_{Rn}}{{\lambda\!\!\:}_{Rn}+{N}_{ac}}.\:$$

Application to the simulated activity:

In contrast to the scenario of Eq. (2), the simulated activity in this work is not constant. As a consequence, the following adapted approach is used for the calculation of the effective dose:

7 $$\begin{aligned} \:{E}_{Rn} &=\underset{\varDelta\:t\:\rightarrow\:0}{\mathrm{lim}}\sum_{k\:=\:0}^{T/\varDelta\:t}\frac{{A}_{{Rn}}\left(k\varDelta\:t\right)}{{V}_{{room}}}\cdot\:{{n}}_{{Rn,ac}}\cdot\:{V}_{{daily}}\cdot\:\varDelta\:t\cdot\:{e}_{{Rn}}\cdot\:F\\ \:\Leftrightarrow{E}_{{Rn}}&={{n}}_{{Rn,ac}}\cdot\:\frac{{V}_{{daily}}}{{V}_{{room}}}\cdot\:{e}_{{Rn}}\cdot\:F\cdot\:{\int\:}_{\!\!\!0\:}^{T}{A}_{{Rn}}\left(t\right)\:dt \\ \:\Leftrightarrow{E}_{{Rn}}&={{n}}_{{Rn,ac}}\cdot\:TI{A}_{Rn,Env}\cdot\:\frac{{V}_{{daily}}}{{V}_{{room}}}\cdot\:{e}_{{Rn}}\cdot\:F, \end{aligned}$$

where *TIA*_*Rn,Env*_ is the time integrated activity (TIA) of ^219^Rn gas in the room air, without consideration of ventilation, over the time span *T*.

The fixed parameters for the calculation of the effective dose are displayed in Table 1. *V*_*room*_, *V*_*daily*_ and *F* are chosen to be the same as in the work of Ooe et al. (2019). However, the effective dose coefficient for ^219^Rn gas was taken from ICRP 137 (ICRP 2017).

**Table 1:** Values for the room volume (*V*_*room*_), the respiration rate (*V*_*daily*_), the exposure factor (*F*), the rate of air changes (*N*_*ac*_), the effective dose coefficient for the inhalation of ^219^Rn gas (*E*_*Rn*_) and the time of exposure (*T*) for the assessment of the effective doses

*V*_*room*_	*V*_*daily*_	*F*	*N*_ac_^*^	*E*_*Rn*_^†^	*T*^‡^
30 m^3^	20 m^3^ d^− 1^	0.5	1 h^− 1^, 6 h^− 1^	4.8∙10^− 11^ Sv Bq^− 1^	15 min, 3 d

^*^In the work of Ooe et al., the rate *N*_*ac*_ = 1 h^− 1^ was used. According to DIN6844-2, the recommended ventilation rate in nuclear medical therapy units in Germany is at least 6 h^− 1^. Both values are used and compared in this work

^†^In this work, the effective dose coefficient for ^219^Rn was taken from ICRP (ICRP 2017)

*V*_*room*_, *V*_*daily*_ and *F* are taken from Ooe et al. (2019)

^‡^Two different time spans *T* for the distinction between the duration of exposure for medical staff (15 min) and caregivers (3 d), are used and compared in this work

According to DIN 6844−2 (DIN 2020), the recommended ventilation rate in nuclear medical therapy units in Germany is at least 6 times per hour. Therefore, the effective dose using Eq. (7) was additionally evaluated accordingly.

### Dosimetry of airborne progeny of ^219^Rn gas

Since ^219^Rn has a relatively short half-life in comparison to ^222^Rn and ^220^Rn, there is limited data available for the calculation of the effective dose caused by inhalation of its airborne progeny. ICRP 137 (ICRP 2017) provides effective dose coefficients only for the inhalation of ^211^Pb and ^211^Bi, based on three assumed aerosol distribution modes for indoor workplaces (see Table 2). For this, the size characteristics for those aerosols are assumed to be equal to those of ^222^Rn progeny. Therefore, the fractions *f*_*i*_ of unattached and attached activity concentrations (ACs)^2^ were also chosen to be equal to those of ^222^Rn progeny for indoor workplaces in this work.

**Table 2:** Effective dose coefficients (Sv Bq^−1^) for inhaled ^211^Pb and ^211^Bi for each mode of assumed aerosol distribution according to ICRP publication 137 (ICRP 2017) and fractions of activity concentrations (AC)

		Effective dose coefficient (Sv Bq^− 1^)	
Mode, *i*	Fraction of AC, *f*_i_^*^	^211^Pb	^211^Bi
Unattached	0.08	6.6∙10^− 8^	4.8∙10^− 9^
Nucleation	0.184	2.2∙10^− 8^	1.5∙10^− 9^
Accumulation	0.736	7.4∙10^− 9^	5.3∙10^− 10^

^*^Since there is no information given on the fractions of AC for the progeny of ^219^Rn in the ICRP Publication 137 (ICRP 2017), in this work, they were assumed to be equal to those of the progeny of ^222^Rn. This has been done for the size characteristics of the modes in the ICRP Publication 137 (ICRP 2017)

In a similar manner as for ^219^Rn gas, the common formula for the effective dose of progeny shown in Eq. (8) is modified.

8 $$\:{E}_{{p}}=F\cdot\:{\sum\:}_{i=1}^{3}{f}_{i}{\sum\:}_{j=1}^{2}\frac{{\bar{A}}_{j}}{{{V}}_{{room}}}\cdot\:{{V}}_{{daily}}\cdot\:T\cdot\:{e}_{ji}$$

By using the ventilation quotient from Eq. (6) and a corresponding quotient for the progeny defined as

9 $$\:{{n}}_{{j,ac}}=\frac{{{\uplambda\:}}\!\!_{j}}{{\lambda\:}\!\!_{j}+{N}_{{ac}}},$$

where *j* stands for the respective progeny, the formula for the effective dose of the airborne progeny of ^219^Rn gas is adapted to

10 $$\begin{aligned} \:{E}_{{p}}=F \cdot\:{{n}}_{{Rn,ac}} \cdot\:{\sum\:}_{i=1}^{3}{f}_{i} \cdot\:{\sum\:}_{j=1}^{2}\frac{{{TIA}}_{j,Env}}{{{V}}_{{room}}} \cdot\:{\prod\:}_{k=j}^{2}{{n}}_{{k,ac}} \cdot\:{{V}}_{{daily}} \cdot\:{e}_{ji}. \end{aligned}$$

In the preceding Eq. (10), *i* represents the mode of assumed aerosol distribution (1 = ‘unattached’, 2 = ‘nucleation’, 3 = ‘accumulation’), *f*_*i*_ stands for the corresponding fraction of AC, *j* represents the considered progeny of ^219^Rn (1 = ‘^211^Bi’, 2 = ‘^211^Pb’), *TIA*_*j,Env*_ is the corresponding TIA of the airborne progeny in the room air over the time span *T* and *e*_*ji*_ is the effective dose coefficient of progeny *j* in mode *i*. The fixed parameters are the same as for ^219^Rn (Table 1). The exposure factor was also used in this calculation (Hosono et al. 2019).

## Results

The results of the simulation are given in activity per infused Bq of ^223^RaCl_2_ (Bq Bq^−1^).

For every comparison of simulation results to real-life applications, if not stated differently, an administration amount of 4015 kBq of ^223^RaCl_2_ is considered. This is the amount for a person with a weight of 73 kg (Bayer 2013), corresponding to a reference adult male according to ICRP (ICRP 2002).

The results of the comparison of the model predicted activities in organs after administration of ^223^Ra to the results of phase-I-studies are documented in the Annex and shortly reviewed in the Discussion.

### Exhaled ^219^Rn gas and airborne progeny

**Fig. 4:**
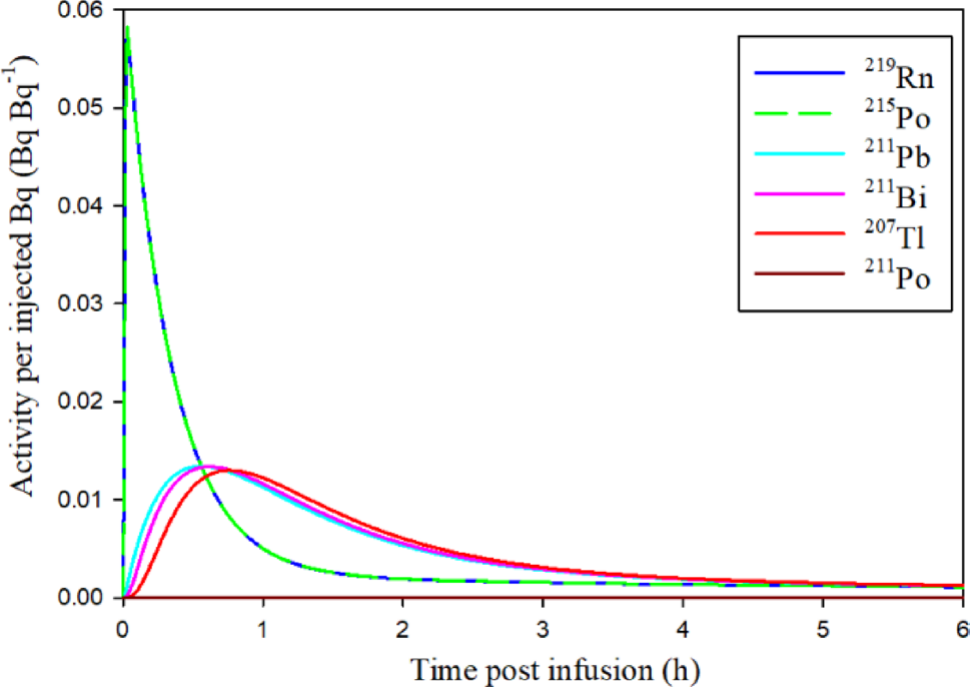
Comparison of activity in the room air for ^219^Rn and its airborne progeny per injected Bq of ^223^RaCl_2_ over a time span of 6 h post infusion

Figure 4 shows activities in the room air per infused Bq of ^223^Ra over a time period of 6 h after the beginning of the infusion for ^219^Rn and its airborne progeny, as simulated in their “Environment” compartments.

The highest activity of ^219^Rn gas is reached at around 1.5 min after the start of the infusion, with an amount of 5.8∙10^− 2^ Bq Bq^− 1^. After that, the activity decreases quite rapidly due to the short half-life of ^219^Rn. After a few hours, ^219^Rn is only found in extremely small amounts in the room air. Assuming the treatment of a reference adult male, the maximum ^219^Rn gas activity found in the room air is 232.9 kBq according to the results of this work.

Due to their very short half-lives, ^219^Rn and ^215^Po rapidly reach secular equilibrium and their activity curves peak at nearly identical values. Because of the very short half-life of ^215^Po and the comparatively long half-life of ^211^Pb, ^211^Pb accumulates with a maximum value of around 1.3∙10^− 2^ Bq Bq^− 1^, which can be seen in the much smoother and lower peak in Fig. 4. The time-activity curves (TACs) of ^211^Pb and ^211^Bi have a very similar shape. Because ^211^Bi has a slightly shorter half-life than ^211^Pb, ^211^Pb and ^211^Bi are in a transient equilibrium and the peak of ^211^Bi reaches approximately 1.3∙10^− 2^ Bq Bq^− 1^. Bismuth-211 decays to ^207^Tl with a probability of more than 99% and ^207^Tl has a half-life twice as long as ^211^Bi. Despite the lower activity concentrations of ^207^Tl during the first 45 min, caused by its longer half-life, a maximum value of 1.3∙10^− 2^ Bq Bq^− 1^ is attained as well. The activity of ^211^Po, on the other hand, is practically negligible due to the very small probability of less than 1% of ^211^Bi decaying to ^211^Po, which can be seen in the TAC of ^211^Po.

**Fig. 5:**
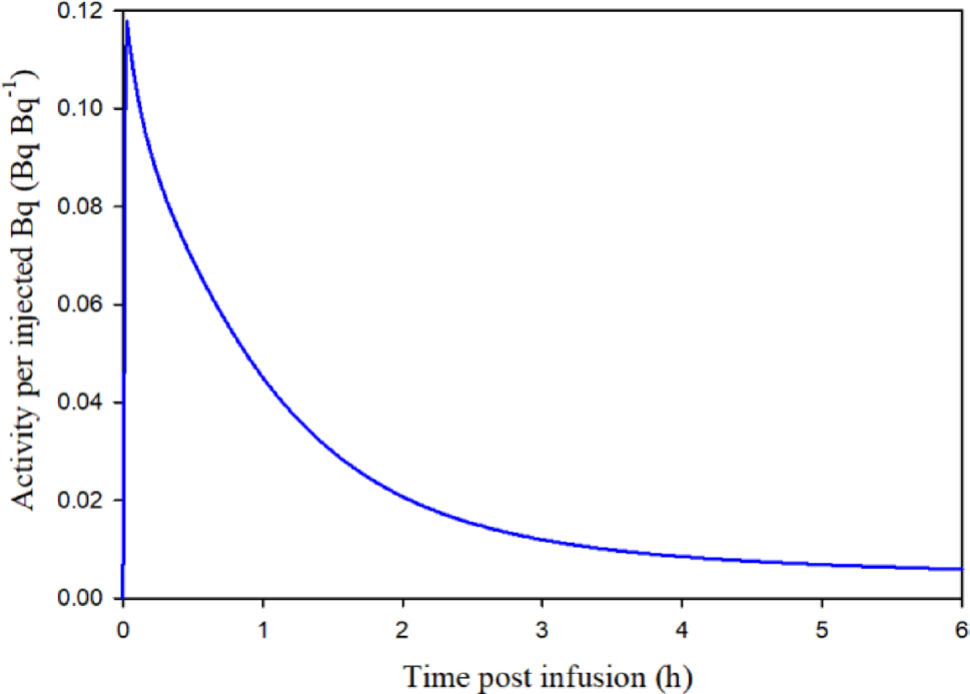
Total activity in the room air as the sum over the activities of ^219^Rn and its airborne progeny per injected Bq of ^223^RaCl_2_ over a time span of 6 h post infusion

The sum over all the activities of ^219^Rn and all its progeny is plotted in Fig. 5 and shows a peak activity of 1.2∙10^− 1^ Bq Bq^− 1^ at around 1.5 min p.i. During the treatment of a reference adult male, this results in a maximum total activity in the room air of approximately 482 kBq. Adding up the activities at the time point of, for example, 30 min p.i. leads to a value of about 6.9∙10^− 2^ Bq Bq^− 1^. In case of treatment of a reference adult male, the activity at this time is estimated to be a total of 278 kBq. At 2 h p.i. the activity in the room air is 84 kBq.

### Comparison of simulated data with literature data

The simulation results for the exhaled activity over an interval of 2.5 s at 1 and 5 min after start of the infusion are displayed in Table 3, together with a comparison to the minimum, mean and maximum exhaled activities per infused Bq acquired from the results of Ooe et al. (2019).

**Table 3:** Comparison of the activity per infused Bq (Bq Bq^−1^) in the patient’s breath at 1 and 5 min post infusion from the model simulation and Ooe et al. (2019)

		Ooe et al. (2019)		
Time	Model simulation	Min	Mean	Max
1 min	2∙10^− 2^	3.7∙10^− 3^	9.7∙10^− 3^	2.4∙10^− 2^
5 min	1.8∙10^− 2^	1.3∙10^− 3^	2.9∙10^− 3^	5.2∙10^− 3^

### ^219^Rn gas transferred from the alimentary tract and its airborne progeny

**Fig. 6:**
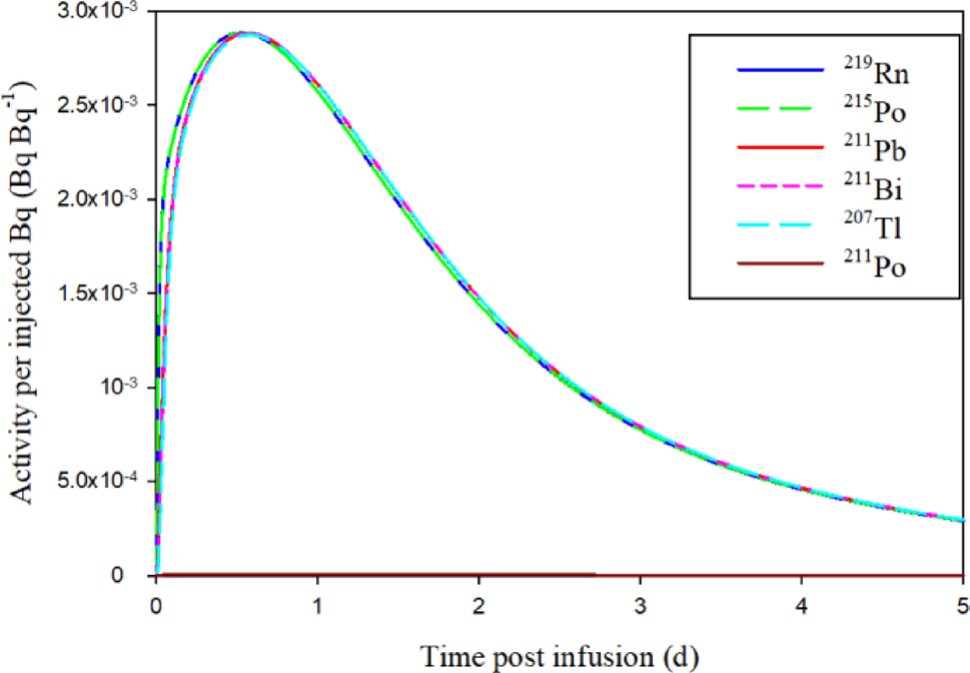
Activity of ^219^Rn from the alimentary tract and of its airborne progeny per infused Bq of ^223^RaCl_2_ over 5 days post infusion

Figure 6 shows the activities of ^219^Rn gas transferred from the alimentary tract to the compartment “Environment 2” and its airborne progeny produced in ”Environment 2”. The TACs follow a similar shape, except for ^211^Po, for which the branching ratio is very low. The peak activity is 2.9∙10^− 3^ Bq Bq^− 1^ for each progeny, except for ^211^Po, resulting in a maximum summed activity of 1.4∙10^− 2^ Bq Bq^− 1^.

### Effective dose due to exhaled ^219^Rn gas and airborne progeny to medical staff and caregivers

To estimate the radiation exposure of medical staff and caregivers, effective doses were calculated for the inhalation of exhaled ^219^Rn gas and its airborne progeny. As before, the patient was considered a reference adult male. The results, under consideration of the two different air change rates *N*_*ac*_ = 1 h^− 1^ and *N*_*ac*_ = 6 h^− 1^, are shown in Table 4 (medical staff) and Table 5 (caregivers).

**Table 4:** Effective doses for inhalation of exhaled ^219^Rn gas and its airborne progeny over 15 min for an administration of 4015 kBq of ^223^RaCl_2_ and two different ventilation rates (*N*_*ac*_)

		Factor for air changes (Π*n*_ac_*)		Effective dose (nSv)	
	TIA (kBq d)	*N*_ac_ = 1 h^− 1^	*N*_ac_ = 6 h^− 1^	*N*_ac_ = 1 h^− 1^	*N*_ac_ = 6 h^− 1^
^219^Rn	1.8	9.98∙10^− 1^	9.91∙10^− 1^	28.7	28.5
^211^Pb	2.5∙10^− 1^	5.3∙10^− 1^	1.6∙10^− 1^	652.5	197
^211^Bi	1.7∙10^− 1^	5.1∙10^− 1^	1.2∙10^− 1^	30.3	7.1
^211^Pb + ^211^Bi				682.8	204.1
^211^Pb + ^211^Bi + ^219^Rn				711.5	232.6

*The factor for the air changes refers to the air change quotients for ^219^Rn (*n*_*Rn,ac*_, ref. to Eq. (6)) and for its airborne progeny (*n*_*Rn,ac*_$\:\cdot\:{\prod\:}_{k = j}^{2}{{n}}_{{k,ac}}$, ref. to Eq. (10))

**Table 5:** Effective doses for inhalation of exhaled ^219^Rn gas and its airborne progeny over 3 days for an administration of 4015 kBq of ^223^RaCl_2_ and two different ventilation rates (*N*_*ac*_)

		Factor for air changes (Π*n*_ac_*)		Effective dose (nSv)	
	TIA (kBq d)	*N*_ac_ = 1 h^− 1^	*N*_ac_ = 6 h^− 1^	*N*_ac_ = 1 h^− 1^	*N*_ac_ = 6 h^− 1^
^219^Rn	9	9.98∙10^− 1^	9.91∙10^− 1^	143.7	142.7
^211^Pb	8.9	5.3∙10^− 1^	1.6∙10^− 1^	23.2∙10^3^	7∙10^3^
^211^Bi	8.9	5.1∙10^− 1^	1.2∙10^− 1^	1.6∙10^3^	373.8
^211^Pb + ^211^Bi				24.8∙10^3^	7.4∙10^3^
^211^Pb + ^211^Bi + ^219^Rn				24.9∙10^3^	7.5∙10^3^

*The factor for the air changes refers to the air change quotients for ^219^Rn (*n*_*Rn,ac*_, ref. to Eq. (6)) and for its airborne progeny (*n*_*Rn,ac*_$\:\cdot\:{\prod\:}_{k = j}^{2}{{n}}_{{k,ac}}$, ref. to Eq. (10))

## Discussion

As a highly volatile gas, ^219^Rn can be exhaled by patients during Xofigo^®^ treatment. Recent publications have demonstrated that a detectable amount of activity can indeed be measured in the room and breathing air of the patients (Ooe et al. 2019; Wanke et al. 2025). Naturally, these measurements are subject to a large uncertainty, due to difficulties in the repeated creation of equal conditions and due to inter-individual variability. A theoretical model predicting the activity exhaled by patients undergoing ^223^RaCl_2_ treatment delivers a useful addition to the research in this field. In this study, the exhalation of ^219^Rn gas was modelled with the help of the biokinetic models provided by ICRP Publication 137 (ICRP 2017). Considering the treatment of a reference adult male, without taking room ventilation into account, this simulation work predicted maximum total activities in the patient’s room air of up to 482 kBq. Nevertheless, the very rapid decline in activity (Fig. 4) gives reassurance that within a few hours, the exposure through exhalation by the patient diminishes quickly.

As shown in the comparison, the model predicted activity concentrations of exhaled ^219^Rn gas after 1 min for an infusion of ^223^RaCl_2_ are within the same order of magnitude as the mean values of the activity concentrations measured by Ooe et al. (2019), even though the simulated values are higher. One possible reason for this could be that the model simulation might overestimate the blood activity levels of ^223^RaCl_2_ in the first few minutes (ref. to the Annex). This would lead to a higher amount of ^219^Rn gas in the blood within the first minutes, resulting in a simulation of increased exhalation of ^219^Rn gas. Additionally, the biokinetic models are based on the first-order linear ODE systems, which are a simplification of the underlying physiological processes. Another contributing reason could be that the models from ICRP 137 (ICRP 2017) describe the biokinetics of a healthy person, whereas all of the patients of Ooe et al. (2019) exhibited bone metastases.

The results of Ooe et al. (2019) revealed a distinctively higher activity during the exhalation after 1 min than after 5 min p.i. Similarly, the model prediction of this work also shows a higher activity for the exhalation after 1 min than after 5 min p.i., however the difference between the two values is not as big as that in the measurements. The measurement results from Ooe et al. (2019) show a large inter-patient variation. Since the one-minute administration of ^223^RaCl_2_ is carried out manually, the time of the peak of exhaled ^219^Rn gas likely varies between the patients, which may account for this variation, as suggested by Ooe et al. (2019). Ooe et al. (2019) assumed that the exhaled ^219^Rn gas activity in the patients breath reaches its peak at approximately 1 min p.i. This suggests that the first minutes after beginning of the infusion are a very sensitive time span, in which small fluctuations in infusion durations, breath sampling times and also sampling durations can considerably change the outcome of the experiment. All these uncertainties are also possible reasons for noticeable differences between the experiment results and the simulation results. Like measurements, simulations are also subject to uncertainties. The biokinetic model for radium provided by ICRP (ICRP 2017) is mostly based on animal data and human accident exposure data. Additonally, the modelling of the exhalation of ^219^Rn is simplified by assuming a direct removal from the blood to the room air. Possible consequential uncertainties have not been accounted for in this work.

In addition to the evaluation of the activity of ^219^Rn gas exhaled by the patient, the corresponding effective dose to the clinical staff and caregivers was assessed and compared to the results of Ooe et al. (2019). Using Eq. (7) and one hourly air change showed a considerably smaller result for the effective dose from inhalation of ^219^Rn gas of 78.2 nSv for medical staff and of 143.7 nSv for caregivers, in comparison to the value of 3.5 µSv estimated by Ooe et al. (2019). This discrepancy can be attributed to two main factors. First of all, the approach of Ooe et al. (2019) is based on the measurement results at 1 min p.i. The equations derived in this study use the integration of the simulated TAC of ^219^Rn in the room air over 15 min and 3 days. The second factor is the different way of inclusion of the ventilation in the treatment room air. In the approach of Ooe et al. (2019), the activity of ^219^Rn gas in the room air was assumed to be removed completely with every hour, without taking the very short half-life of ^219^Rn gas of less than 4 s into account. In this work, the impact of the decay constant of ^219^Rn on the factor for the air changes in the treatment room is taken into consideration. The increase of the hourly air changes from 1 h^− 1^ to 6 h^− 1^, according to German regulations, does not yield a significant change in the result for the effective dose from inhalation of ^219^Rn. Because of the short half-life of ^219^Rn, the two factors for the air changes are very similar and close to 1 (see Tables 4 and 5). However, in the case of the progeny of ^219^Rn, the increase of the hourly air changes has a large impact on the results for the effective doses. Comparing the results for the effective dose between clinical staff and caregivers, it can be seen that approximately 3% of the radiation exposure occurs within the first 15 min after beginning of the infusion. Considering the short half-lives of the respective isotopes, it is clear that the highest exposure risk lies within the first hours after infusion of ^223^RaCl_2_ (ref. to Figs. 4 and 5). Due to the lack of dosimetric information on ^219^Rn and its progeny, an accurate value for the effective dose of the progeny remains challenging.

From the results of this work, it can be assumed that the highest amounts of ^219^Rn gas is exhaled by the patient within the first two hours p.i. After 24 h, the amount will strongly diminish. Therefore, caution should be taken during the first hours after beginning of the application of ^223^RaCl_2_.

Compared to the activity in the room air caused by exhalation, the activity of ^219^Rn leaving the patient’s alimentary tract and of its airborne progeny is of extremely low value. Therefore, the radiation exposure caused by this should not be a major concern.

In the comparison to the clinical patient data of the phase-I-studies in the Annex, the radium model described in ICRP Publication 137 (ICRP 2017) shows some deviations. In general, it can be expected that an ICRP population model, which was initially developed for healthy people, shows different biokinetics than a person with bone metastases, at least in some parts. Since ^223^Ra is a bone seeking calcium mimetic (Parker et al. 2013), it can be assumed that bone metastases are likely to take up amounts of ^223^RaCl_2_ (Xofigo^®^), that would have been found elsewhere in the body. However the modelled TACs show very similar behaviours as the clinical data points of the phase-I-studies. Although the model predicted activities do not always match the mean or median values evaluated in the studies, they often remain within the measured ranges. Especially the activities in blood, whole body, intestine and the cumulative activity in urine and faeces are represented quite well in the model simulation. Activities in bones, liver and kidneys showed noticeable differences in the results of the simulation in comparison to the phase-I-studies. Considering the fact, that there is not yet a pharmacokinetic model specifically for ^223^RaCl_2_, the radium model of ICRP (ICRP 2017) delivers a good basis for the development of the model structure.

## Conclusion

In this study, biokinetic modelling was used to predict the amount of radioactivity of ^219^Rn gas and its airborne progeny in the breathing air of the patients and the room air after the beginning of the Xofigo^®^ treatment. The modelled exhalation of ^219^Rn gas at 1 min p.i. in this study is comparable to the measured values reported by literature data. Based on the modelled activities, the effective dose from inhalation of ^219^Rn gas and its airborne progeny was calculated to be 232.6 nSv for medical staff and 7.5 µSv caregivers.

## Supplementary Information

Below is the link to the electronic supplementary material.

Supplementary material 1 (DOCX 16.6 kb)

## Figures and Tables

**Fig. 7:**
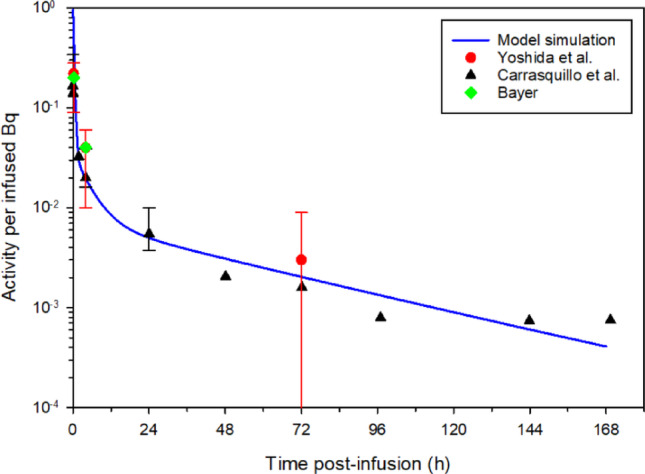
Activity of ^223^RaCl_2_ in the blood per infused Bq of ^223^RaCl_2_: Comparison of the result from the model simulation (blue) to the results from Yoshida et al. (2016) (red), Carrasquillo et al. (2013) (black) and the data from Bayer (2013) (green) over a time span of 7 d

**Fig. 8:**
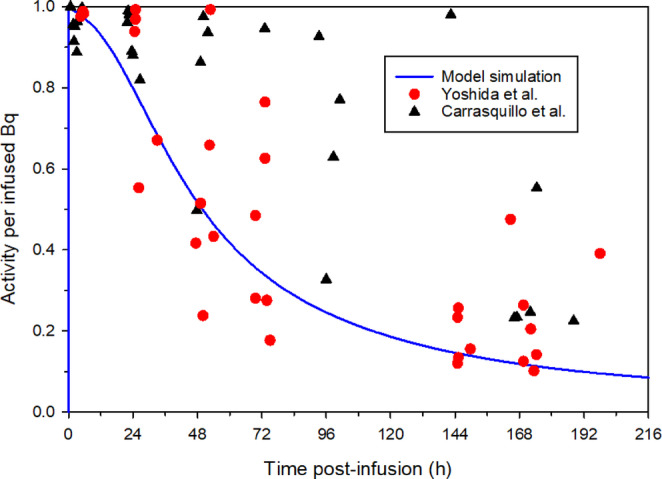
Activity of ^223^RaCl_2_ in the whole body per infused Bq of ^223^RaCl_2_: Comparison of the result from the model simulation (blue) to the results from Yoshida et al. (2016) (red) and Carrasquillo et al. (2013) (black) over a time span of 9 d

**Fig. 9:**
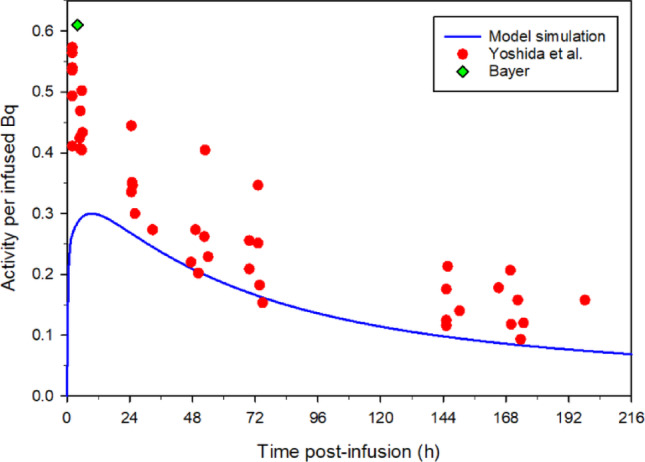
Activity of ^223^RaCl_2_ in the bones per infused Bq of ^223^RaCl_2_: Comparison of the result from the model simulation (blue) to the results from Yoshida et al. (2016) (red) and the data point from Bayer (2013) (green) over a time span of 9 d

**Fig. 10:**
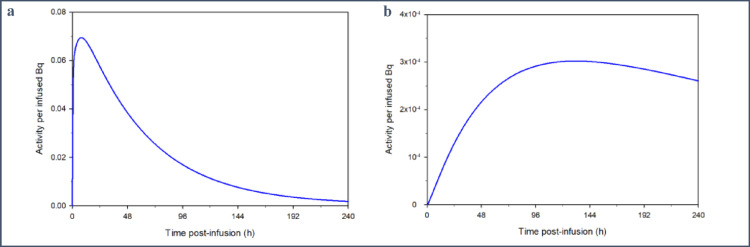
Activity of ^223^RaCl_2_ in the liver per infused Bq of ^223^RaCl_2_ over a time span of 10 d according to the model simulation: **a** activity in the whole liver (compartments Liver 1 and Liver 2 summed up) and **b** activity in compartment Liver 2 separately

**Fig. 11:**
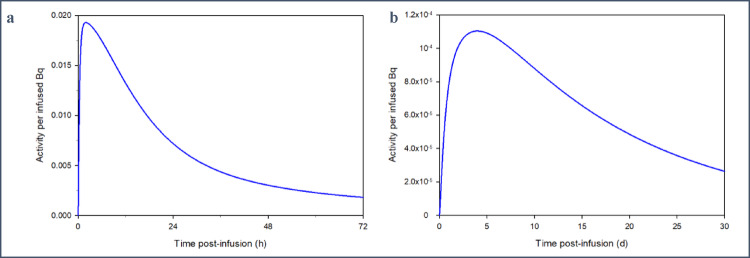
Activity of ^223^RaCl_2_ in the kidneys per infused Bq of ^223^RaCl_2_ according to the model simulation: **a** activity in the whole kidneys (compartments Kidneys 1 and Kidneys 2 summed up) over a time span of 3 d and **b** activity in compartment Kidneys 2 separately over a time span of 30 d

**Fig. 12:**
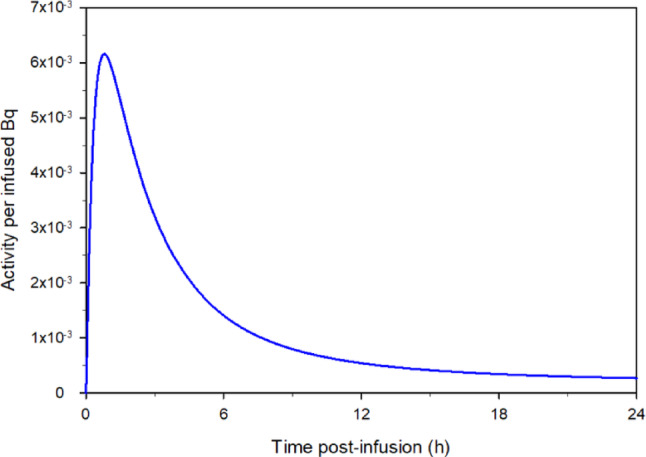
Activity of ^223^RaCl_2_ in the bladder per infused Bq of ^223^RaCl_2_ over a time span of 24 h

**Fig. 13:**
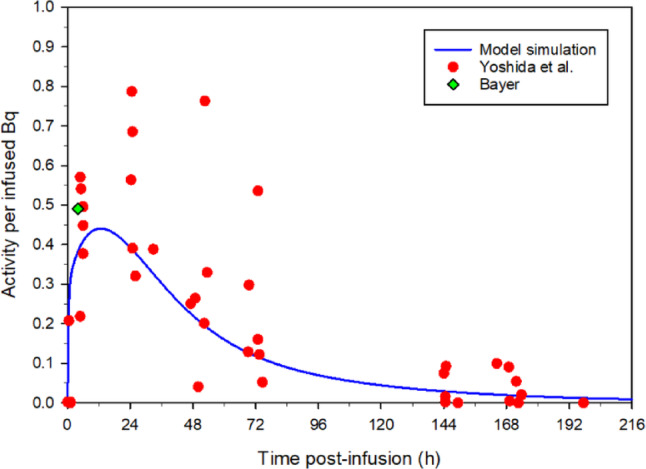
Activity of ^223^RaCl_2_ in the large intestine per infused Bq of ^223^RaCl_2_ : Comparison of the result from the model simulation (blue) to the results from Yoshida et al. (2016) (red) and the data from Bayer (2013) (green) over a time span of 9 d

**Fig. 14:**
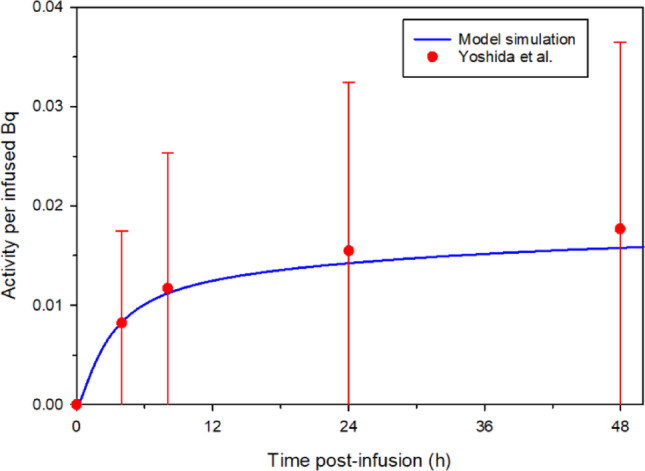
Cumulative activity of ^223^RaCl_2_ in urine per infused Bq of ^223^RaCl_2_ : Comparison of the result from the model simulation (blue) to the results from Yoshida et al. (red) (2016) over a time span of 2 d

**Fig. 15:**
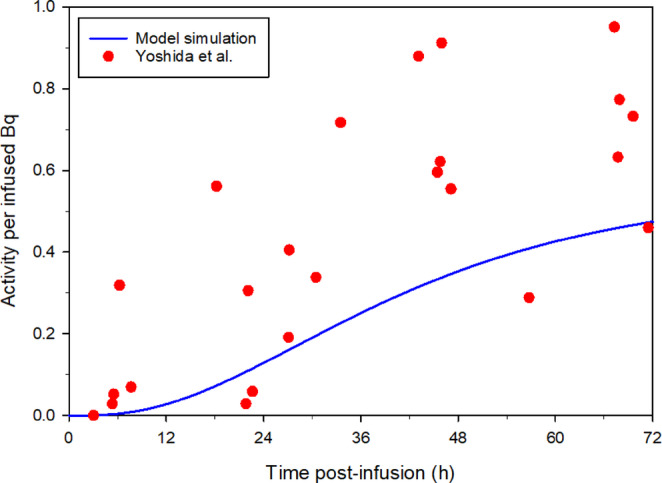
Cumulative activity of ^223^RaCl_2_ in faeces per infused Bq of ^223^RaCl_2_: Comparison of the result from the model simulation (blue) to the results from Yoshida et al. (red) (2016) over a time span of 3 d

## Data Availability

No datasets were generated or analysed during the current study.

## Annex – Comparison of model predicted biokinetics of Radium-223 in the human body post-injection with phase-I clinical studies of Xofigo^®^

For the comparisons conducted in this annex, the results from phase I studies of Yoshida et al. (2016) and Carrasquillo et al. (2013), as well as the data provided in the prescribing information for Xofigo^®^ (Bayer 2013), were examined. If results were not explicitly stated but only depicted in graphs, the online data extraction tool PlotDigitizer (https://plotdigitizer.com/app) was used to obtain numerical values. For the Bayer (Bayer 2013) comparison, an infusion time of 1 min was applied for the model simulation. For the phase-I-studies of Yoshida et al. (2016) and Carrasquillo et al. (2013), where infusion times ranged between 2 and 5 min, a 2-minute infusion time was selected for the simulation. It is important to note that choosing an infusion time between 2 and 5 min primarily influences the results only in the initial minutes following the infusion and the impact is minimal.

### Blood

The activity levels of ^223^Ra in the blood reveal a similar behaviour across all phase-I-studies considered in this comparison (Yoshida et al. 2016; Carrasquillo et al. 2013; Bayer 2013), demonstrating a rapid clearance from the blood. Although the model simulation overestimates the blood activity levels in the first few minutes, it quickly aligns with the values reported in the studies, indicating that the rapid clearance from the blood is appropriately modelled. This initial overestimation may be attributed to slightly underestimated transfer parameters from the blood to other compartments in the model of ^223^RaCl_2_. The comparison is illustrated in Fig. 7 and the corresponding data points are listed in Table 6.

**Table 6:** Comparison of the activity fraction of ^223^Ra in the blood in the work of Yoshida et al. (2016), Carrasquillo et al. (2013) and Bayer (2013) to the results of the model simulation of this work. Values are given as median, mean or range. Every number was rounded to one decimal place

Time p.i. (h)	Yoshida et al.	Carrasquillo et al.	Bayer	Model simulation
0	-	1.7∙10^− 1^	-	0
End of infusion	-	1.4∙10^− 1^ (9∙10^− 2^ − 3.4∙10^− 1^)	-	9.5∙10^− 1^
2.5∙10^− 1^	2.2∙10^− 1^ (9∙10^− 2^ − 2.8∙10^− 1^)	-	2∙10^− 1^	5∙10^− 1^
1.8	-	3.2∙10^− 2^	-	3.1∙10^− 2^
4	4∙10^− 2^ (1∙10^− 2^ − 6∙10^− 2^)	2∙10^− 2^ (1.6∙10^− 2^ − 3.9∙10^− 2^)	4∙10^− 2^	2∙10^− 2^
24	-	5.5∙10^− 3^ (3.7∙10^− 3^ − 1∙10^− 2^)	-	5∙10^− 3^
48.1	-	2∙10^− 3^	-	3.1 ∙10^− 3^
72	3∙10^− 3^ (0–9∙10^− 3^)	-	-	2 ∙10^− 3^
72.2	-	1.6∙10^− 3^	-	2 ∙10^− 3^
97	-	7.9∙10^− 4^	-	1.3 ∙10^− 3^
143.8	-	7.4∙10^− 4^	-	6.1 ∙10^− 4^
169.4	-	7.5∙10^− 4^	-	4 ∙10^− 4^

### Whole body

The measurements of Carrasquillo et al. (2013) and Yoshida et al. (2016) describe the same clearance patterns for the whole-body activity as the implementation of this work. The comparison to Carrasquillo et al. (2013) shows similarities in activity fractions throughout the first day, but afterwards, the values of the simulation fall below the measurements. As for Yoshida et al. (2016), the outcomes of the simulation lie completely within the range of the measurements. Overall, the biokinetic model from ICRP Publication 137 (ICRP 2017) seems to represent the whole-body clearance quite well. The comparison is illustrated in Fig. 8 and the corresponding data points are listed in Table 7.

**Table 7:** Comparison of the activity fraction of ^223^Ra in the whole body in the work of Yoshida et al. (2016) and Carrasquillo et al. (2013) to the results of the model simulation of this work. Values are given as median or mean. Every number was rounded to one decimal place

Yoshida et al.			Carrasquillo et al.		
**Time p.i. (h)**	**Patient 1**	**Model simulation**	**Time p.i. (h)**	**Patient 1**	**Model simulation**
5.7	9.8∙10^− 1^	9.7∙10^− 1^	6.7∙10^− 1^	1	1
24.9	9.9∙10^− 1^	7.9∙10^− 1^	3.4	9.6∙10^− 1^	9.8∙10^− 1^
49.2	5.2∙10^− 1^	5.1∙10^− 1^	22.2	9.9∙10^− 1^	8.2∙10^− 1^
69.6	4.8∙10^− 1^	3.6∙10^− 1^	45.8	1	5.4∙10^− 1^
144.9	2.3∙10^− 1^	1.4∙10^− 1^	101	7.7∙10^− 1^	2.3∙10^− 1^
172.2	0.2	1.1∙10^− 1^	174.4	5.5∙10^− 1^	1.1∙10^− 1^
**Time p.i. (h)**	**Patient 2**	**Model simulation**	**Time p.i. (h)**	**Patient 2**	**Model simulation**
0	1	0	6.7∙10^− 1^	1	1
24.9	9.7∙10^− 1^	7.9∙10^− 1^	22.6	9.8∙10^− 1^	8.2∙10^− 1^
52.8	9.9∙10^− 1^	4.7∙10^− 1^	49.2	8.6∙10^− 1^	5.1∙10^− 1^
73.2	7.6∙10^− 1^	3.4∙10^− 1^	73.1	9.5∙10^− 1^	3.4∙10^− 1^
145.2	2.6∙10^− 1^	1.4∙10^− 1^	142.4	9.8∙10^− 1^	1.5∙10^− 1^
169.5	2.6∙10^− 1^	1.2∙10^− 1^			
**Time p.i. (h)**	**Patient 3**	**Model simulation**	**Time p.i. (h)**	**Patient 3**	**Model simulation**
0	1	0	1.7	9.6∙10^− 1^	9.9∙10^− 1^
4.5	9.8∙10^− 1^	9.8∙10^− 1^	3	8.9∙10^− 1^	9.8∙10^− 1^
24.6	9.4∙10^− 1^	7.9∙10^− 1^	26.6	8.2∙10^− 1^	7.7∙10^− 1^
54	4.3∙10^− 1^	4.6∙10^− 1^	51.9	9.4∙10^− 1^	4.8∙10^− 1^
69.6	2.8∙10^− 1^	3.6∙10^− 1^	98.7	6.3∙10^− 1^	2.4∙10^− 1^
149.7	1.6∙10^− 1^	1.4∙10^− 1^	172.1	2.5∙10^− 1^	1.1∙10^− 1^
174.3	1.4∙10^− 1^	1.1∙10^− 1^			
**Time p.i. (h)**	**Patient 4**	**Model simulation**	**Time p.i. (h)**	**Patient 4**	**Model simulation**
0	1	0	5.1	1	1
5.4	9.9∙10^− 1^	9.7∙10^− 1^	23.9	8.8∙10^− 1^	0.8
33	6.7∙10^− 1^	6.8∙10^− 1^	47.5	1	0.5
47.4	4.2∙10^− 1^	5.2∙10^− 1^	167	0.2	0.1
73.8	2.8∙10^− 1^	3.3∙10^− 1^			
145.2	1.3∙10^− 1^	1.4∙10^− 1^			
169.5	1.2∙10^− 1^	1.2∙10^− 1^			
**Time p.i. (h)**	**Patient 5**	**Model simulation**	**Time p.i. (h)**	**Patient 7**	**Model simulation**
0	1	0	2	9.1∙10^− 1^	9.9∙10^− 1^
25.5	1	7.8∙10^− 1^	23.6	8.9∙10^− 1^	0.8
52.5	6.6∙10^− 1^	4.8∙10^− 1^	47.8	0.5	5.2∙10^− 1^
73.2	6.3∙10^− 1^	3.4∙10^− 1^	96	3.3∙10^− 1^	2.5∙10^− 1^
164.7	4.8∙10^− 1^	1.2∙10^− 1^	166	2.3∙10^− 1^	1.2∙10^− 1^
198	3.9∙10^− 1^	0.1			
**Time p.i. (h)**	**Patient 6**	**Model simulation**	**Time p.i. (h)**	**Patient 9**	**Model simulation**
0	1	0	2.4	9.5∙10^− 1^	9.9∙10^− 1^
5.4	9.9∙10^− 1^	9.7∙10^− 1^	21.9	9.6∙10^− 1^	8.2∙10^− 1^
26.1	5.5∙10^− 1^	7.7∙10^− 1^	50.2	9.8∙10^− 1^	0.5
50.1	2.4∙10^− 1^	0.5	93.3	9.3∙10^− 1^	2.5∙10^− 1^
75	1.8∙10^− 1^	3.3∙10^− 1^	188.2	2.2∙10^− 1^	0.1
144.9	1.2∙10^− 1^	1.4∙10^− 1^			
173.4	0.1	1.1∙10^− 1^			

### Bones

Activity levels in the bones, similar to those overserved in the whole body, are lower in the simulation compared to the findings of Yoshida et al. (2016), however, the TAC from the simulation aligns well with the trend observed in the measurement results. At 4 h post-infusion, Bayer (2013) reports an activity concentration that is double the amount the biokinetic model predicts. This underestimation by the model may be attributed to the fact that the model of ICRP Publication 137 (ICRP 2017) describes the biokinetics of radium for a reference worker without accounting for the presence of bone metastases. However, in the study of Yoshida et al. (2016), each of the participants has more than two scintigraphically confirmed bone metastases. Due to the high chemical similarities of radium to calcium, individuals with bone metastases are likely to have a higher uptake of ^223^RaCl_2_ in regions carrying bone metastases, which is the therapeutic target with Xofigo^®^. The comparison details are illustrated in Fig. 9 and the corresponding data points are listed in Table 8.

**Table 8:** Comparison of the activity fraction of ^223^Ra in the bones in the work of Yoshida et al. (2016) and Bayer (2013) to the results of the model simulation of this work. Values are given as median or mean. Every number was rounded to one decimal place

Yoshida et al.			Yoshida et al.		
**Time p.i. (h)**	**Patient 1**	**Model simulation**	**Time p.i. (h)**	**Patient 2**	**Model simulation**
2	4.9∙10^− 1^	2.7∙10^− 1^	2	5.7∙10^− 1^	2.7∙10^− 1^
5.9	4.3∙10^− 1^	2.9∙10^− 1^	5.6	0.5	2.9∙10^− 1^
24.8	3.5∙10^− 1^	2.7∙10^− 1^	24.6	4.4∙10^− 1^	2.7∙10^− 1^
49.1	2.7∙10^− 1^	2.1∙10^− 1^	52.8	0.4	0.2
69.7	2.6∙10^− 1^	1.7∙10^− 1^	73.1	3.5∙10^− 1^	1.6∙10^− 1^
145.1	1.8∙10^− 1^	0.1	145.7	2.1∙10^− 1^	0.1
172.5	1.6∙10^− 1^	8.4∙10^− 2^	169.7	2.1∙10^− 1^	8.5∙10^− 2^
**Time p.i. (h)**	**Patient 3**	**Model simulation**	**Time p.i. (h)**	**Patient 4**	**Model simulation**
2	5.4∙10^− 1^	2.7∙10^− 1^	2	5.4∙10^− 1^	2.7∙10^− 1^
4.8	4.2∙10^− 1^	2.9∙10^− 1^	5.6	0.4	2.9∙10^− 1^
24.6	3.4∙10^− 1^	2.7∙10^− 1^	32.8	2.7∙10^− 1^	2.4∙10^− 1^
53.9	2.3∙10^− 1^	0.2	47.4	2.2∙10^− 1^	2.1∙10^− 1^
69.7	2.1∙10^− 1^	1.7∙10^− 1^	73.7	1.8∙10^− 1^	1.6∙10^− 1^
150.2	1.4∙10^− 1^	9.5∙10^− 2^	145.1	1.2∙10^− 1^	0.1
174.8	1.2∙10^− 1^	8.3∙10^− 2^	170	1.2∙10^− 1^	8.5∙10^− 2^
**Time p.i. (h)**	**Patient 5**	**Model simulation**	**Time p.i. (h)**	**Patient 6**	**Model simulation**
2	4.1∙10^− 1^	2.7∙10^− 1^	2	5.6∙10^− 1^	2.7∙10^− 1^
5.1	4.1∙10^− 1^	2.9∙10^− 1^	5.1	4.7∙10^− 1^	2.9∙10^− 1^
25.1	3.5∙10^− 1^	2.7∙10^− 1^	26	0.3	2.6∙10^− 1^
52.5	2.6∙10^− 1^	0.2	50.3	0.2	0.2
73.1	2.5∙10^− 1^	1.6∙10^− 1^	74.8	1.5∙10^− 1^	1.6∙10^− 1^
165.2	1.8∙10^− 1^	8.7∙10^− 2^	145.1	1.2∙10^− 1^	0.1
198.2	1.6∙10^− 1^	7.4∙10^− 2^	173.6	9.3∙10^− 2^	8.4∙10^− 2^

Bayer					
**Time p.i. (h)**	**Activity concentration**	**Model simulation**			
4	6.1∙10^− 1^	2.8∙10^− 1^			

### Liver

The phase-I-studies (Yoshida et al. 2016; Carrasquillo et al. 2013; Bayer 2013) indicate only mild uptake of activity in the liver, primarily occurring within the first 4 h after infusion. In contrast, this work’s simulation shows a peak activity fraction of nearly 7% at approximately 8 h post-infusion, with levels remaining above 1% for the first 5 d. As shown in Fig. A4b, the compartment “Liver 2” contributes minimally to these values, suggesting that the higher activity is predominantly localized in the compartment “Liver 1” (see Fig. 10a).

### Kidneys and bladder

Similarly, the activity in kidneys appears to be higher in the simulation compared to the measurements of Carrasquillo et al. (2013) and the results provided in the prescribing information (Bayer 2013). The phase-I-studies (Yoshida et al. 2016; Carrasquillo et al. 2013) reported a rapid clearance and no significant amounts of activity after 4 h. In contrast, the simulation indicates nearly 2% of the injected activity still present in the kidneys at 4 h. It is only around 17 h post-infusion that the activity fraction falls below 1% of the injected activity. This higher activity is primarily attributed to the compartment “Kidneys 1” (see Fig. 11).

The activity in the bladder seems to be modelled accurately, since the simulation, similar to the findings from Bayer (2013) and Carrasquillo et al. (2013), results in values of less than 1%. This is demonstrated in Fig. 12

### Intestine

In the large intestine, Carrasquillo et al. (2013) did not observe any activity during the first hours. At 24 h, they were able to measure 52% of the infused activity. Bayer (2013) reports a fraction of 49% of injected activity in the large bowel at 4 h post-injection. Although the simulation shows activity in the large intestine with a rapid increase from the start, it reports approximately 39% activity at both 4 and 24 h, which is lower than the results from the phase I studies. While the simulation may slightly underestimate intestinal activity, its results are still within the range observed by Yoshida et al. (2016). The radium model from ICRP Publication 137 (ICRP 2017) does not account for the small intestine in the biodistribution. However, both Carrasquillo et al. (2013) and Yoshida et al. (2016) reported activity in the small intestine during the first 24 h. Therefore, incorporating the small intestine into the biokinetic modelling of ^223^RaCl_2_ could be beneficial. The comparison is illustrated in Fig. 13, with the corresponding data points detailed in Table 9.

**Table 9:** Comparison of the activity fraction of ^223^Ra in the large intestine in the work of Yoshida et al. (2016) and Bayer (2013) to the results of the model simulation of this work. Values are given as median or mean. Every number was rounded to one decimal place

Yoshida et al.			Yoshida et al.		
**Time p.i. (h)**	**Patient 1**	**Model simulation**	**Time p.i. (h)**	**Patient 2**	**Model simulation**
1.1	0	3.1∙10^− 1^	1.1	2.3∙10^− 3^	3.1∙10^− 1^
6	3.8∙10^− 1^	4.1∙10^− 1^	6	4.5∙10^− 1^	4.1∙10^− 1^
24.9	6.9∙10^− 1^	3.9∙10^− 1^	24.3	5.6∙10^− 1^	3.9∙10^− 1^
48.9	2.6∙10^− 1^	2.2∙10^− 1^	52.6	7.6∙10^− 1^	1.9∙10^− 1^
69.4	0.3	1.3∙10^− 1^	72.9	5.4∙10^− 1^	1.2∙10^− 1^
144	7.4∙10^− 2^	2.9∙10^− 2^	144.9	9.3∙10^− 2^	2.9∙10^− 2^
171.7	5.4∙10^− 2^	1.8∙10^− 2^	168.9	9∙10^− 2^	1.9∙10^− 2^
**Time p.i. (h)**	**Patient 3**	**Model simulation**	**Time p.i. (h)**	**Patient 4**	**Model simulation**
5.7∙10^− 1^	2.1∙10^− 1^	2.5∙10^− 1^	1.1	2.3∙10^− 3^	3.1∙10^− 1^
4.9	5.7∙10^− 1^	3.9∙10^− 1^	6	0.5	4.1∙10^− 1^
24.6	7.9∙10^− 1^	3.9∙10^− 1^	32.9	3.9∙10^− 1^	3.3∙10^− 1^
53.4	3.3∙10^− 1^	1.9∙10^− 1^	47.1	2.5∙10^− 1^	2.3∙10^− 1^
69.1	1.3∙10^− 1^	1.3∙10^− 1^	73.4	1.2∙10^− 1^	1.1∙10^− 1^
149.4	0	2.7∙10^− 2^	144.6	2.3∙10^− 3^	2.9∙10^− 2^
173.7	2∙10^− 2^	1.8∙10^− 2^	169.1	4.5∙10^− 3^	1.9∙10^− 2^
**Time p.i. (h)**	**Patient 5**	**Model simulation**	**Time p.i. (h)**	**Patient 6**	**Model simulation**
0	2.3∙10^− 3^	0	1.1	0	3.1∙10^− 1^
4.9	2.2∙10^− 1^	3.9∙10^− 1^	5.1	5.4∙10^− 1^	0.4
24.9	3.9∙10^− 1^	3.9∙10^− 1^	26	3.2∙10^− 1^	3.8∙10^− 1^
52.3	0.2	0.2	50	4.1∙10^− 2^	2.1∙10^− 1^
72.9	1.6∙10^− 1^	1.2∙10^− 1^	74.6	5.2∙10^− 2^	1.1∙10^− 1^
164.3	0.1	2.1∙10^− 2^	144.6	1.6∙10^− 2^	2.9∙10^− 2^
197.4	0	1.2∙10^− 2^	172.6	0	1.8∙10^− 2^

**Bayer**					
**Time p.i. (h)**	**Activity concentration**	**Model simulation**			
4	4.9∙10^− 1^	3.8∙10^− 1^			

### Urine and faeces

Regarding the elimination of ^223^Ra from the body, the results from Carrasquillo et al. (2013) indicate a median total excretion of 76%, which is equal to the fraction calculated from the model simulation. However, the simulated total activity in urinary excretion is only half of the measured median reported by Carrasquillo et al. (2013) and the information provided by Bayer (2013). In contrast, the simulated cumulative activity in urine closely matches the fitted plot of the arithmetic mean of Yoshida et al. (2016). The comparison is illustrated in Fig. 14, with the corresponding data points listed in Table 10.

**Table 10:** Comparison of the activity fraction of ^223^Ra in the urine in the work of Yoshida et al. (2016) to the results of the model simulation of this work. Values are given as median or mean and range. The times p.i. were rounded to one decimal place and value was rounded to one decimal place

Time p.i. (h)	Yoshida et al.	Model simulation
0	0	0
4	8.2∙10^− 3^ (0–1.7∙10^− 2^)	8.3∙10^− 3^
8	1.2∙10^− 2^ (0–2.5∙10^− 2^)	1.1∙10^− 2^
24	1.5∙10^− 2^ (0–3.2∙10^− 2^)	1.4∙10^− 2^
48	1.8∙10^− 2^ (0–3.6∙10^− 2^)	1.6∙10^− 2^

The model simulation underestimates the cumulative activity in faecal excretion, but it mostly stays within the range of the measurements of Yoshida et al. (2016) (see Fig. 15; Table 11).

**Table 11:** Comparison of the activity fraction of ^223^Ra in the faeces in the work of Yoshida et al. (2016) to the results of the model simulation of this work. Values are given as median or mean. Every number was rounded to one decimal place

Yoshida et al.			Yoshida et al.		
**Time p.i. (h)**	**Patient 1**	**Model simulation**	**Time p.i. (h)**	**Patient 2**	**Model simulation**
3	0	5.2∙10^− 4^	22.6	5.9∙10^− 2^	1.1∙10^− 1^
45.4	0.6	3.3∙10^− 1^	56.7	2.9∙10^− 1^	4.1∙10^− 1^
67.7	6.3∙10^− 1^	4.6∙10^− 1^			
**Time p.i. (h)**	**Patient 3**	**Model simulation**	**Time p.i. (h)**	**Patient 4**	**Model simulation**
5.5	5.2∙10^− 2^	3.3∙10^− 3^	5.3	2.8∙10^− 2^	3∙10^− 3^
30.5	3.4∙10^− 1^	1.9∙10^− 1^	7.7	6.9∙10^− 2^	8.3∙10^− 3^
45.8	6.2∙10^− 1^	3.4∙10^− 1^	22.1	3.1∙10^− 1^	1.1∙10^− 1^
67.9	7.7∙10^− 1^	4.6∙10^− 1^	47.1	5.5∙10^− 1^	3.5∙10^− 1^
			69.6	7.3∙10^− 1^	4.7∙10^− 1^
**Time p.i. (h)**	**Patient 5**	**Model simulation**	**Time p.i. (h)**	**Patient 6**	**Model simulation**
21.8	3∙10^− 2^	1.1∙10^− 1^	6.2	3.2∙10^− 1^	4.6∙10^− 3^
27.1	1.9∙10^− 1^	1.6∙10^− 1^	18.2	5.6∙10^− 1^	7.3∙10^− 2^
27.2	4.1∙10^− 1^	1.6∙10^− 1^	33.5	7.2∙10^− 1^	2.3∙10^− 1^
71.4	4.6∙10^− 1^	4.7∙10^− 1^	43.1	8.8∙10^− 1^	3.1∙10^− 1^
			46	9.1∙10^− 1^	3.4∙10^− 1^
			67.3	9.5∙10^− 1^	4.6∙10^− 1^

## Footnotes

1 The exposure factor takes the duration of the contact and the distance from the patient during the time of exposure into consideration. Therefore, there are different exposure factors defined for different degrees of involvement.

2 The fractions of unattached and attached activity concentrations (AC) were taken from ICRP Publication 137 (ICRP 2017), where they are defined as fractions of potential alpha energy concentration (PAEC).
