# Supplementary material for: Biokinetic modelling of the exhalation of 219Rn gas and its airborne progeny from patients undergoing treatment with 223Ra-dichloride and effective dose estimation for caregivers
Source: Radiat Environ Biophys. 2026 May 18;65(2):741–56. doi: 10.1007/s00411-025-01193-5 (PMC13303660; doi:10.1007/s00411-025-01193-5)
Supplement: Supplementary file 1 — Supplementary material 1 (DOCX 16.6 kb) [file 411_2025_1193_MOESM1_ESM.docx]

**Supplementary material**

**Table S1** Transfer coefficients for radium (ICRP 2017, 2006, 1995)

| From | To | Transfer coefficient (d^-1^) |
| --- | --- | --- |
| Blood | Other soft tissues 0 | 20.93 |
| Blood | Other soft tissues 1 | 3.5 |
| Blood | Other soft tissues 2 | 0.07 |
| Blood | Cortical bone surface | 7.78 |
| Blood | Trabecular bone surface | 9.72 |
| Blood | Kidneys 1 | 1.4 |
| Blood | Urinary bladder content | 0.606 |
| Blood | Liver 1 | 4.2 |
| Blood | Right colon content | 21.79 |
| Other soft tissues 0 | Blood | 6.98 |
| Other soft tissues 1 | Blood | 0.693 |
| Other soft tissues 2 | Blood | 0.00038 |
| Cortical bone surface | Blood | 0.578 |
| Cortical bone surface | Exch. cortical bone volume | 0.116 |
| Exch. cortical bone volume | Cortical bone surface | 0.0185 |
| Exch. cortical bone volume | Non-exch. cortical bone volume | 0.0046 |
| Non-exch. cortical bone volume | Blood | 0.0000821 |
| Trabecular bone surface | Blood | 0.578 |
| Trabecular bone surface | Exch. trabecular bone volume | 0.116 |
| Exch. trabecular bone volume | Trabecular bone surface | 0.0185 |
| Exch. trabecular bone volume | Non-exch. trabecular bone volume | 0.0046 |
| Non-exch. trabecular bone volume | Blood | 0.000493 |
| Kidneys 1 | Blood | 2.073 |
| Kidneys 1 | Kidneys 2 | 0.00624 |
| Kidneys 2 | Blood | 0.0019 |
| Urinary bladder content | Urine | 12 |
| Liver 1 | Blood | 0.691 |
| Liver 1 | Liver 2 | 0.00208 |
| Liver 2 | Blood | 0.0019 |
| Right colon content | Left colon content | 2 |
| Left colon content | Rectosigmoid | 2 |
| Rectosigmoid | Faeces | 2 |

Exch., exchangeable; non-exch., non-exchangeable.

**Table S2** Transfer coefficients for radon as a progeny of radium (ICRP 2017, 1995)

| From | To | Transfer coefficients (d^-1^) |
| --- | --- | --- |
| Other soft tissues 0 | Blood | 33.27106 |
| Other soft tissues 1 | Blood | 33.27106 |
| Other soft tissues 2 | Blood | 33.27106 |
| Cortical bone surface | Blood | 100 |
| Exch. cortical bone volume | Blood | 1.5 |
| Non-exch. cortical bone volume | Blood | 0.36 |
| Trabecular bone surface | Blood | 100 |
| Exch. trabecular bone volume | Blood | 1.5 |
| Non-exch. trabecular bone volume | Blood | 0.36 |
| Kidneys 1 | Blood | 33.27106 |
| Kidneys 2 | Blood | 33.27106 |
| Urinary bladder content | Urine | 12 |
| Liver 1 | Blood | 33.27106 |
| Liver 2 | Blood | 33.27106 |
| Right colon content | Environment 2 | 100 |
| Left colon content | Environment 2 | 100 |
| Rectosigmoid | Environment 2 | 100 |
| Blood | Environment | 1000 |

Exch., exchangeable; non-exch., non-exchangeable.
